# Negative effects of diabetes–related distress on health-related quality of life: an evaluation among the adult patients with type 2 diabetes mellitus in three primary healthcare clinics in Malaysia

**DOI:** 10.1186/s12955-015-0384-4

**Published:** 2015-11-24

**Authors:** Boon-How Chew, Sherina Mohd-Sidik, Sazlina Shariff-Ghazali

**Affiliations:** Department of Family Medicine, Faculty of Medicine and Health Sciences, Universiti Putra Malaysia, 43400 Serdang, Selangor Malaysia; Department of Psychiatry, Faculty of Medicine and Health Sciences, Universiti Putra Malaysia, Serdang, 43400 Selangor Malaysia

**Keywords:** Quality of life, Distress, Depression, Type 2 diabetes mellitus, Primary care, Religious beliefs

## Abstract

**Background:**

Patients with type 2 diabetes mellitus (T2D) often experienced change in life, altered self-esteem and increased feelings of uncertainty about the future that challenge their present existence and their perception of quality of life (QoL). There was a dearth of data on the association between diabetes-related distress (DRD) and health-related quality of life (HRQoL). This study examined the determinants of HRQoL, in particular the association between DRD and HRQoL by taking into account the socio-demographic-clinical variables, including depressive symptoms (DS) in adult patients with T2D.

**Methods:**

This cross-sectional study was conducted in 2012–2013 in three public health clinics in Malaysia. The World Health Organization Quality of Life-Brief (WHOQOL-BREF), 17-items Diabetes Distress Scale (DDS-17), and 9-items Patient Health Questionnaire (PHQ-9) were used to measure HRQoL, DRD and DS, respectively. The aim of this research was to examine the association between the socio-demographic-clinical variables and HRQoL as well as each of the WHOQOL-BREF domain score using multivariable regression analyses.

**Results:**

The response rate was 93.1 % (700/752). The mean (SD) for age was 56.9 (10.18). The majority of the patients were female (52.8 %), Malay (53.1 %) and married (79.1 %). About 60 % of the patients had good overall HRQoL. The mean (SD) for Overall QoL, Physical QoL, Psychological QoL, Social Relationship QoL and Environmental QoL were 61.7 (9.86), 56.7 (10.64), 57.9 (11.73), 66.8 (15.01) and 65.3 (13.02), respectively. The mean (SD) for the total DDS-17 score was 37.1 (15.98), with 19.6 % (136/694) had moderate distress. DDS-17 had a negative association with HRQoL but religiosity had a positive influence on HRQoL (B ranged between 3.07 and 4.76). Women, especially younger Malays, who had diabetes for a shorter period of time experienced better HRQoL. However, patients who were not married, had dyslipidaemia, higher levels of total cholesterol and higher PHQ-9 scores had lower HRQoL. Macrovascular complications showed the largest negative effect on the overall HRQoL (adjusted B = −4.98, 95 % CI −8.56 to −1.40).

**Conclusion:**

The majority of primary care adult with T2D had good overall HRQoL. Furthermore, the independent determinants for HRQoL had also concurred with many past studies. In addition, the researchers found that DRD had negative effects on HRQoL, but religiosity had positive influence on HRQoL. Appropriate support such as primary care is needed for adult patients with T2D to improve their life and their HRQoL.

**Trial registration:**

NMRR-12-1167-14158

## Background

Type 2 diabetes mellitus (T2D) has been known to have changed life experiences, altered self-esteem, challenged present existence and increased uncertainty about the future [1–8].

The lives of individuals would change from the moment when they experience the symptoms such as chronic hyperglycaemia, weight loss, skin infections and lethargy right up to the diagnosis of diabetes mellitus. A complete change in life routines continues from the demands of more regular healthy life-styles, adherence to daily medication and scheduled visits to various types of healthcare professionals. Repeated physical examinations, laboratory tests and psychosocial counselling reverberating the ideas of risky threats to bodily health and the long-term complications [9] could reduce the self-esteem and self-confidence that inspire adventures and motivation in the patients’ lives [10, 11]. Family members and friends would also avoid discussing the implications of the disease with the patients [12, 13]. There might be occasional feelings of stigmatisation at family gatherings or even at work-related social functions [7, 14]. Left alone to these physical and psychological onslaughts, many patients may feel overwhelmed, shaken and questioned their personal values and beliefs of the purpose of self-regulation [10], self-care and self-management [15]. Hope for the future may be replaced with dread of complications from the disease and the adverse effects of medications which may result in frequent and disruptive effects such as restlessness, distress, anxiety, and depression [3, 16]. Eventually, all of these negative emotions may lead to the failure of adherence to health recommendations, medications, medical follow-ups [17, 18], and may increase the number of medical leaves [16]. Consequently, patients’ personal perceptions turn from bad to worse, and they start to smoke excessively [19], practise uncontrolled diet, and have conflicts with their significant others [20]. This would cause the symptoms of diabetes and their associated complications to return despite escalating their medications [21].

Quality of life (QoL) of the patients with diabetes mellitus represents personal perceptions of life experience, social, vocational and domestic functioning against hope and ideals from aspects of physical, psychological, relationships, environmental and spiritual domains [22, 23]. A large body of literature has focused on the impacts of diabetes mellitus of both type 1 and type 2 on patients, albeit in vast aspects and contexts of life [4, 21, 24], and the associations with QoL, self-efficacy and disease control for adult patients with T2D. Moreover, patients who perceived higher levels of QoL also showed that they had better social support, acceptance of the seriousness and consequences of the disease, and had less difficulty in managing their diabetes [25]. In spite of the many different QoL scales used in past studies, there are similar effects of socio-demographic variables and clinical parameters on the domains of QoL across the globe [18, 26–34]. However, there was a dearth of data on the association between diabetes-related distress (DRD) and health-related quality of life (HRQoL). Accordingly, the purpose of this study was to evaluate the determinants of HRQoL, particularly to examine the effects of DRD on HRQoL taking into account other socio-demographic-clinical variables of adult patients with T2D who received regular primary medical care in three public health clinics in Malaysia. Hence, the research question was: what are the independent effects of socio-demographic-clinical variables, including DRD and depressive symptoms (DS), on HRQoL in adult patients with T2D?

## Methods

### Ethics, consent and permission

This cross-sectional study was approved by the Medical Research Ethics Committee (MREC), Malaysia’s Ministry of Health with the reference number NMRR-12-1167-14158. Respondents had provided their written informed consent before participating in the study, and the anonymity of the patients was preserved throughout the study.

### Setting

Participants were recruited from three public health clinics: Seri Kembangan Health Clinic (SK), Dengkil Health Clinic (DK) and Salak Health Clinic (SL) in Malaysia. These health clinics were chosen because they were different in terms of the patients’ characteristics and geographical regions. SK is an urban clinic located in the vicinity of the Chinese community, and thus it is visited mainly by Chinese patients. DK is a rural clinic frequented mostly by Indians in a larger proportion than a usual public health clinic. SL is a rural clinic situated in a Malay-majority residential area. The variability of the sites provided a broad range of patients on which the associations of DRD, HRQoL and socio-demographic clinical parameters can be assessed. The purpose of this report was to determine the associations of the independent variables and the dependent variables irrespective of the health clinics.

### Patient

#### Definitions of study participants

The researchers had sampled consecutively all the patients with T2D who came to the clinics. The patients were at least 30 year-old and were diagnosed with T2D for more than a year. The patients who were recruited for this study fulfilled the following criteria: have been on regular follow-ups, had at least three visits at the clinic in the past 1 year and had recent blood test results done within the past 3 months. Patients who were pregnant or lactating, had psychiatric/psychological disorders that could impair judgment and memory, and could not read or understand English, Malay or Mandarin were excluded from this study. Patients who fulfilled the criteria were approached and informed of the study and their written consents were obtained before the questionnaires were administered in their preferred language. Trained research assistants also interviewed the patients who were not able to answer the questionnaires themselves.

Besides a questionnaire on demography (age, gender, ethnicity, religion, educational level, occupation, monthly income, self-perceive social support) and smoking status, a structured case record form was used to document the patients’ history which includes co-morbidities (hypertension and hyperlipidaemia/dyslipidaemia), diabetes-related complications, duration of the diabetes, HbA1c, blood pressure, lipid profiles, number and type of medication used. Three questionnaires were distributed to evaluate HRQoL, DRD and DS, and they were prepared in three languages: English, Malay and Mandarin.

#### Definitions of diseases

The definition of T2D was when their case records fulfilled any of these criteria: (i) either having documented a diagnosis of diabetes mellitus according to the 1999 World Health Organization criteria [35] or (ii) currently treated with lifestyle modifications, oral anti-hyperglycaemic agents or insulin. Hypertension was diagnosed if the systolic blood pressure was ≥ 130 mm Hg and/or the diastolic blood pressure was ≥ 80 mm Hg on two of the successive readings obtained by the clinic’s physicians. A blood pressure (BP) < 130/80 mmHg was regarded as controlled BP, and this was the mean of the two readings in the rested position with the arm positioned at the heart level, using a cuff of an appropriate size. Hyperlipidaemia refers to an increase in the concentration of one or more plasma or serum lipids, usually caused by cholesterol and triglycerides, and the term dyslipidaemia was used for either an increase or decrease in the concentration of one or more plasma or serum lipids [a low density lipoprotein-cholesterol (LDL-C) > 2.6 mmol/L, triglyceride (TG) > 1.7 mmol/L and high density lipoprotein-cholesterol (HDL-C) < 1.1 mmol/L]. Body mass index (BMI) was calculated as weight was divided by height squared. A LDL-C ≤ 2.6 mmol/L and HbA1c ≤ 6.5 % were regarded as the other treatment targets [36, 37]. These clinical data were retrieved from the patient’s medical records using a case record form on the same day when the patient completed the questionnaires.

#### Definitions of diabetes-related complications

There were five diabetes-related complications in this study. Three were classified as microvascular complications (MicroCx) which comprised retinopathy, nephropathy, and diabetic foot problems (DFP). Another two complications were classified as macrovascular complications (MacroCx) which comprised ischemic heart disease and cerebrovascular disease, or stroke. These complications were retrieved from the patient’s medical records. Diagnoses of these complications were made or confirmed by the attending physicians at the clinics based on the medical symptoms, laboratory results, radiological evidence, and treatment history obtained from the clinic and other hospitals. Nephropathy was diagnosed if any of the following was present: microalbuminuria, proteinuria, serum creatinine > 150 mmol/L or estimated glomerular filtration rate < 60mls/min (calculated using Cockroft-Gault formula) persisted (≥2 occasions with at least 3 months apart). DFP comprised foot deformity, current ulcer, amputation, peripheral neuropathy, or peripheral vascular disease.

### Instruments

#### Health-related quality of life

The World Health Organization Quality of Life- Brief (WHOQOL-BREF) was chosen in this study as the HRQoL measure. Although it is not diabetes-specific, it is relevant to people with diabetes [22]. The WHOQOL-BREF was developed internationally, cross-culturally and produces four HRQoL domains [38]. The four domain scores denote an individual’s perception of HRQoL in the following domains: Physical (PQOL), Psychological (YQOL), Social Relationships (SRQOL) and Environment (EQOL). The sum of these domain scores produces the overall quality of life (OQOL) [39]. This four-domain structure has the comparative fit index of 0.901, which demonstrates a good internal consistency with Cronbach alpha values for each of the four domain scores which ranged from 0.66 (for domain 3) to 0.84 (for domain 1) [39, 40]. There are also two items on overall perceptions of HRQoL that are examined separately. Question 1 asks about the individual’s overall perception of HRQoL, and question 2 asks about the individual’s overall perception of his or her health.

Each item, from item number 3 to 26, contributes equally to their domain score. After the negatively framed constituent questions were reverse scored, domain scores were calculated by computing the mean of the item scores within the domain. The mean domain scores were then multiplied by 4 providing a range of 4 to 20 and transformed to 100 ([score – 4] * 100/16) in further analyses. The sum of the domain scores were scaled in a positive direction (i.e. higher scores denote higher HRQoL). Where up to two items were missing, the mean of the other items in the domain were substituted. Where more than 20 % of the data were missing from an assessment, the assessment was discarded. Where more than two items were missing from the domain, the domain score was not calculated (with the exception of domain 3, where the domain was only calculated if < 1 item was missing). There were high correlations between the domain scores based on the WHOQOL-100 and domain scores calculated using items in the WHOQOL-BREF [39]. These correlations ranged from 0.89 (for domain 3) to 0.95 (for domain 1). The WHOQOL-BREF was shown to be comparable to the WHOQOL-100 which could discriminate between the ill and well groups [38, 39].

#### Diabetes-Related Distress (DRD)

The 17-item Diabetes Distress Scale (DDS-17) assesses problems and hassles concerning diabetes in the past 1 month on a Likert scale which had measurements of 1 (not a problem) to 6 (a very serious problem) [41]. The total DDS-17 score ranges from 17 to 102. The score was calculated by summing up the patient’s responses to the appropriate items and divided by the number of items in that scale. A mean item score of ≥ 3 (moderate distress) is considered a level of distress worthy of clinical attention. The DDS-17 has been found to have adequate and better psychometric properties compared to other similar scales [42]. A local translation and validation study of the Malay version of DDS-17 showed a high internal consistency (Cronbach’s α = 0.94) and test–retest reliability value of 0.33 (*P* = 0.009) [43]. There was a significant relationship between the mean DDS-17 item score categories (<3 vs ≥ 3) and HbA1c categories (<7 % vs ≥ 7 %) (*X*^2^ = 4.20; *P* = 0.048). The DDS-17 sensitivity and specificity, with positive and negative predictive values were 56.5, 23.8, 7.6 and 83.1 %, respectively. A Chinese version of the DDS-17 was found to have good psychometric properties with Cronbach’s α of 0.90 for internal consistency and test–retest reliability coefficient of 0.74 [44].

#### Depressive Symptoms (DS)

The Patient Health Questionnaire (PHQ-9) has been known to have a good construct and criterion validity in making diagnosis and assessing the severity of DS, of which a total score of ≥ 10 indicates a sensitivity of 88 % and a specificity of 89 % for major DS [45]. The PHQ-9 refers to the symptoms experienced by patients during the 2 weeks prior to answering the questionnaire (for example thoughts that you would be better off dead or of hurting yourself in some way). The PHQ-9 scores range from 0 to 27, and each of the nine items has a score starting from 0 (not at all) to 3 (nearly every day). PHQ-9 scores of 0–4, 5–9, 10–14, 15–19, and 20–27 represent none to minimal, mild, moderate, moderately severe, and severe DS, respectively. The Malay version of PHQ-9 had been locally validated with a sensitivity of 87 % (95 % confidence interval 71 to 95 %), a specificity of 82 % (74 to 88 %), positive likelihood ratio (LR) 4.8 (3.2, 7.2) and negative LR 0.16 (0.06, 0.40) [46]. DS was included in this study as one of the main covariates of DRD [47]. The Chinese version of the PHQ-9 is reported to have good psychometric properties with an internal consistency of 0.82 and test–retest reliability of 0.76 over a 2-week interval [48].

### Statistical analyses

A sample size was calculated using GPower 3.1.2 software with an estimated effect size at 1.5 [47, 49], of DRD on HRQoL, with a power of 0.95 and significance at 0.05; the estimated sample size was 500. Taking into consideration the fact that 30 % of the data in the patient’s medical records were either incomplete or missing, and 30 % of the data in the questionnaires filled by the patients were incomplete, and the sample size needed was 650.

Quantitative data analyses were then executed with IBM SPSS Statistics version 21.0. Comparisons of mean levels were performed using the Student’s t-test for unpaired samples and Chi square test was used for proportionate samples. If the value of *P* < 0.05, it was considered to be significant at two tails. Independent variables include the demographic, DRD (measured by DDS-17), DS (measured by PHQ-9), and clinical data.

The researchers had observed that the socio-demographic characteristic was according to the tertile of the overall quality of life (OQOL). Visual inspections on the histogram and the statistical tests confirmed the normal distributions of OQOL and the four domain scores (PQOL, YQOL, SRQOL and EQOL). Then the relationship between the independent variables towards OQOL and each of the domain score were examined using multiple linear regression analyses.

Univariable analyses were executed for all the studied variables and the variables that showed significant effects on the WHOQOL-BREF scores were included in the final multiple linear regression analyses. Socio-demographic data that were continuous and ordinal were entered in the linear regression while the nominal variables were transformed to binary (such as ethnicity, religion and marital status). Employment status variable was re-ordered into three categories: unemployed (0), retired/home manager (1) and employed (2) based on the initial assessments of the direction of the effect on WHOQOL-BREF scores. Clinical variables that had intervals were first entered in the linear regression. The absence of association prompted the entry of the clinical variables to be in a binary nature in accordance to the recommended targets of control (1). MicroCx and MacroCx were preferentially entered in sequence in the linear regression analyses instead of the combined variable of any diabetes complications which was only entered when both the MicroCx and MacroCx were found not in association with HRQoL. This was done so that the individual effects of MicroCx or MacroCx could be discovered, and the combined variable for both of these complications would confirm the true state of no association with a larger sample size.

The multicollinearity between the variables was checked with a correlation matrix and an inspection of their standard error (SE) magnitude. In this study, no variables correlated with each other, *r* < 0.2 and SEs were all within 0.001 to 5.0. In all the final models, Q-Q plots gave an indication of normality, the residual plots indicated a fulfilment of linearity and homogeneity assumptions. Age, gender and ethnicity were included in all the models because these three variables were potential confounders.

## Results

The participants’ response rate was 93.1 % (700/752). Out of these respondents, 694, 700, 697 and 698 had complete data on summed OQOL, PQOL, YQOL, SRQOL and EQOL, respectively. The mean (SD) for age was 56.9 (10.18) years. More than half were women (52.8 %) and Malay (53.1 %) (Table 1). Furthermore, the majority of the respondents were either married or living with partners (79.1 %), had non-tertiary education (89.0 %), and were earning < RM 3000 (about USD 850) per month (94.4 %). Most patients did some exercise (57.6 %) and never smoked (76.1 %). About 80 % of the patients reported that they had hypertension compared to about 40 % who had dyslipidaemia. Patients who used oral hypoglycaemic (OHA), anti-hypertensive agents (AHA), and lipid-lowering agents (LLA) were about 91 %, 88 % and 77 %, respectively (Table 1).

**Table 1:** The socio-demographic and clinical characteristics according to the tertile of the World Health Organization Quality of Life- Brief (WHOQOL-BREF)

		Tertile WHOQoL-BREF total score, *n* (%)				*X* ^2^	*P* ^*^
		Total^a^	Low^b^	Intermediate^b^	High^b^		
Health Clinic	Seri Kembangan	218 (31.4)	79 (36.2)	75 (34.4)	64 (29.4)	12.40	0.015
	Dengkil	123 (17.7)	43 (35.0)	50 (40.7)	30 (24.4)		
	Salak	353 (50.9)	107 (30.3)	107 (30.3)	139 (39.4)		
Age, year	≤50	186 (26.9)	62 (33.3)	52 (28.0)	72 (38.7)	5.90	0.206
	51–60	268 (38.7)	90 (33.6)	88 (32.8)	90 (33.6)		
	>60	238 (34.4)	76 (31.9)	91 (38.2)	71 (29.8)		
Diabetes Duration, year	<5	341 (50.8)	111 (32.6)	106 (31.1)	124 (36.4)	8.61	0.072
	5–9.9	187 (27.9)	58 (31.0)	60 (32.1)	69 (36.9)		
	≥10	143 (21.3)	52 (36.4)	57 (39.9)	34 (23.8)		
Gender	Female	365 (52.8)	120 (32.9)	127 (34.8)	118 (32.3)	0.79	0.673
	Male	326 (47.2)	106 (32.5)	105 (32.2)	115 (35.3)		
Ethnicity	Malay	365 (53.1)	109 (29.9)	120 (32.9)	136 (37.3)	16.48	0.002
	Chinese	160 (23.3)	73 (45.6)	46 (28.8)	41 (25.6)		
	Indian	163 (23.7)	46 (28.2)	62 (38.0)	55 (33.7)		
Religion	No religion	32 (4.6)	13 (40.6)	10 (31.3)	9 (28.1)	26.22	0.003
	Moslem	373 (53.9)	109 (29.2)	124 (33.2)	140 (37.5)		
	Buddhist	81 (11.7)	45 (55.6)	19 (23.5)	17 (21.0)		
	Hinduism/Sikh	147 (21.2)	41 (27.9)	57 (38.8)	49 (33.3)		
	Christian/Catholic	22 (3.2)	7 (31.8)	7 (31.8)	8 (36.4)		
	Others	37 (5.3)	13 (35.1)	14 (37.8)	10 (27.0)		
Religiosity	Religious	590 (85.5)	183 (31.0)	197 (33.4)	210 (35.6)	11.95	0.018
	Unsure	24 (3.5)	13 (54.2)	9 (37.5)	2 (8.3)		
	Not religious	76 (11.0)	31 (40.8)	25 (32.9)	20 (26.3)		
Marital status	Married/living with a partner	546 (79.1)	176 (32.2)	184 (33.7)	186 (34.1)	4.69	0.584
	Widowed	97 (14.1)	30 (30.9)	35 (36.1)	32 (33.0)		
	Divorced/separated	21 (3.0)	11 (52.4)	4 (19.0)	6 (28.6)		
	Single	26 (3.8)	10 (38.5)	8 (30.8)	8 (30.8)		
Education	Never	45 (6.6)	18 (40.0)	16 (35.6)	11 (24.4)	7.15	0.128
	Primary & secondary	563 (82.4)	185 (32.9)	195 (34.6)	183 (32.5)		
	Tertiary	75 (11.0)	22 (29.3)	19 (25.3)	34 (45.3)		
Employment	Retired/home manager	368 (53.3)	127 (34.5)	131 (35.6)	110 (29.9)	7.64	0.106
	Employed	312 (45.2)	95 (30.4)	98 (31.4)	119 (38.1)		
	Unemployed	11 (1.6)	6 (54.5)	2 (18.2)	3 (27.3)		
Income (RM)	<1000	323 (47.4)	113 (35.0)	101 (31.3)	109 (33.7)	3.32	0.506
	1000–2999	320 (47.0)	99 (30.9)	115 (35.9)	106 (33.1)		
	≥3000	38 (5.6)	9 (23.7)	14 (36.8)	15 (39.5)		
Exercise	No	293 (42.5)	112 (38.2)	91 (31.1)	90 (30.7)	10.94	0.027
	≤3 times in a week	231 (33.5)	77 (33.3)	79 (34.2)	75 (32.5)		
	>3 times in a week	166 (24.1)	39 (23.5)	60 (36.1)	67 (40.4)		
Smoking	Never	526 (76.1)	177 (33.7)	175 (33.3)	174 (33.1)	5.14	0.273
	Stop > 5 years	60 (8.7)	20 (33.3)	14 (23.3)	26 (43.3)		
	Stop ≤ 5 years and active smoker	105 (15.2)	32 (30.5)	41 (39.0)	32 (30.5)		
BMI, Kgm^−2^	<23.0	76 (11.2)	32 (42.1)	22 (28.9)	22 (28.9)	7.75	0.101
	23.0–27.4	237 (35.0)	82 (34.6)	82 (34.6)	73 (30.8)		
	≥27.5	365 (53.8)	103 (28.1)	125 (34.2)	137 (37.5)		
HbA1c, %	HbA1c ≥ 6.5	515 (83.3)	168 (32.6)	171 (33.2)	176 (34.2)	0.26	0.879
	HbA1c <6.5	103 (16.7)	36 (35.0)	34 (33.0)	33 (32.0)		
	HbA1c ≥ 7.0	455 (73.6)	148 (32.5)	153 (33.6)	154 (33.8)	0.23	0.892
	HbA1c <7.0	163 (26.4)	56 (34.4)	52 (31.9)	55 (33.7)		
Hypertension	No	148 (21.7)	46 (31.1)	45 (30.4)	57 (38.5)	1.71	0.426
	Yes	534 (78.3)	180 (33.7)	179 (33.5)	175 (32.8)		
BP, mmHg	BP > 130/80	479 (69.5)	155 (32.4)	155 (32.4)	169 (35.3)	1.53	0.466
	BP ≤130/80	210 (30.5)	72 (34.3)	74 (35.2)	64 (30.5)		
Dyslipidaemia	No	406 (60.9)	124 (30.5)	125 (30.8)	157 (38.7)	9.94	0.007
	Yes	261 (39.1)	95 (36.4)	96 (36.8)	70 (26.8)		
LDL-C, mmol/L	LDL-C > 2.6	336 (59.9)	106 (31.5)	120 (35.7)	110 (32.7)	2.72	0.257
	LDL-C ≤ 2.6	225 (40.1)	74 (32.9)	66 (29.3)	85 (37.8)		
HDL-C, mmol/L	HDL-C < 1.1	407 (72.2)	118 (29.0)	143 (35.1)	146 (35.9)	5.86	0.053
	HDL-C ≥ 1.1	157 (27.8)	62 (39.5)	45 (28.7)	50 (31.8)		
TG, mmol/L	TG > 1.7	250 (44.3)	81 (32.4)	85 (34.0)	84 (33.6)	0.36	0.837
	TG ≤1.7	314 (55.7)	99 (31.5)	102 (32.5)	113 (36.0)		
Total-C, mmol/L	Total-C > 4.5	357 (58.1)	121 (33.9)	126 (35.3)	110 (30.8)	4.81	0.090
	Total-C ≤ 4.5	257 (41.9)	78 (30.4)	78 (30.4)	101 (39.3)		
Any diabetes complication	No	608 (87.7)	193 (31.7)	201 (33.1)	214 (35.2)	6.31	0.043
	Yes	85 (12.3)	36 (42.4)	30 (35.3)	19 (22.4)		
Microvascular complication	No	641 (92.5)	207 (32.3)	212 (33.1)	222 (34.6)	4.23	0.120
	Yes	52 (7.5)	22 (42.3)	19 (36.5)	11 (21.2)		
Macrovascular complication	No	648 (93.9)	206 (31.8)	217 (33.5)	225 (34.7)	6.92	0.031
	Yes	42 (6.1)	21 (50.0)	13 (31.0)	8 (19.0)		
OHA	No	60 (8.7)	25 (41.7)	20 (33.3)	15 (25.0)	3.03	0.220
	Yes	630 (91.3)	202 (32.1)	210 (33.3)	218 (34.6)		
Insulin	No	422 (61.2)	132 (31.3)	139 (32.9)	151 (35.8)	2.27	0.687
	1 type	187 (27.1)	66 (35.3)	64 (34.2)	57 (30.5)		
	2 types	80 (11.6)	29 (36.3)	26 (32.5)	25 (31.3)		
Number of AHA agents	No	81 (11.8)	22 (27.2)	30 (37.0)	29 (35.8)	4.71	0.581
	1 type	204 (29.6)	70 (34.3)	65 (31.9)	69 (33.8)		
	2 types	203 (29.5)	73 (36.0)	59 (29.1)	71 (35.0)		
	3 types	201 (29.2)	62 (30.8)	75 (37.3)	64 (31.8)		
LLA	No	156 (22.6)	50 (32.1)	47 (30.1)	59 (37.8)	1.59	0.451
	Yes	533 (77.4)	177 (33.2)	182 (34.1)	174 (32.6)		
APA	No	612 (89.1)	194 (31.7)	202 (33.0)	216 (35.3)	6.48	0.039
	Yes	75 (10.9)	32 (42.7)	27 (36.0)	16 (21.3)		

^*^Chi-square *P* value

*BP* blood pressure, *LDL-C* low-density lipoprotein cholesterol, *HDL-C* high-density lipoprotein cholesterol, *TG* triglycerides, *Total-C* total cholesterol, *AHA* anti-hypertensive agent, *BMI* body mass index, *BP* blood pressure, *APA* anti-platelet agent, *OHA* oral hypoglycaemic agent, *LDL-C* low density lipoprotein- cholesterol, *HDL-C* high density lipoprotein- cholesterol, *LLA* lipid-lowering agent, *RM* Ringgit Malaysia, *TG* triglyceride, *Total-C* total-cholesterol, *WHOQoL-BREF* World Health Organization Quality of Life-Brief 26 items

^a^Column percentage

^b^Row percentage

There were only a minority of patients who achieved treatment targets. About 10 % attained BMI < 23 kgm^−2^, 15 % achieved HbA1c < 6.5 %, 25 % achieved HbA1c < 7.0 %, 30 % attained BP ≤ 130/80 mmHg and 40 % attained LDL-C ≤ 2.6 mml/L (Table 1). In addition, about 12 % had one of the diabetes-related complications, and this rate seemed to parallel with the number of patients who were on anti-platelet agents (Table 1).

**Fig. 1:**
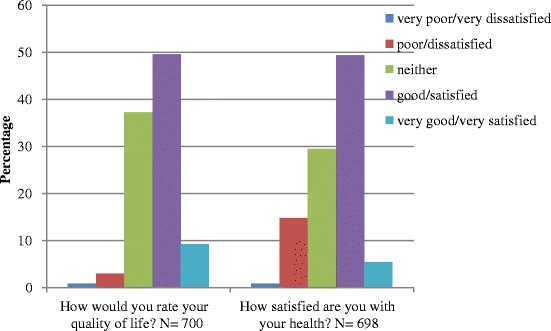
The proportion of responses to the first two items of the WHOQOL-BREF on the overall perception of health. WHOQOL-BREF= World Health Organization Quality of Life- Brief

**Fig. 2:**
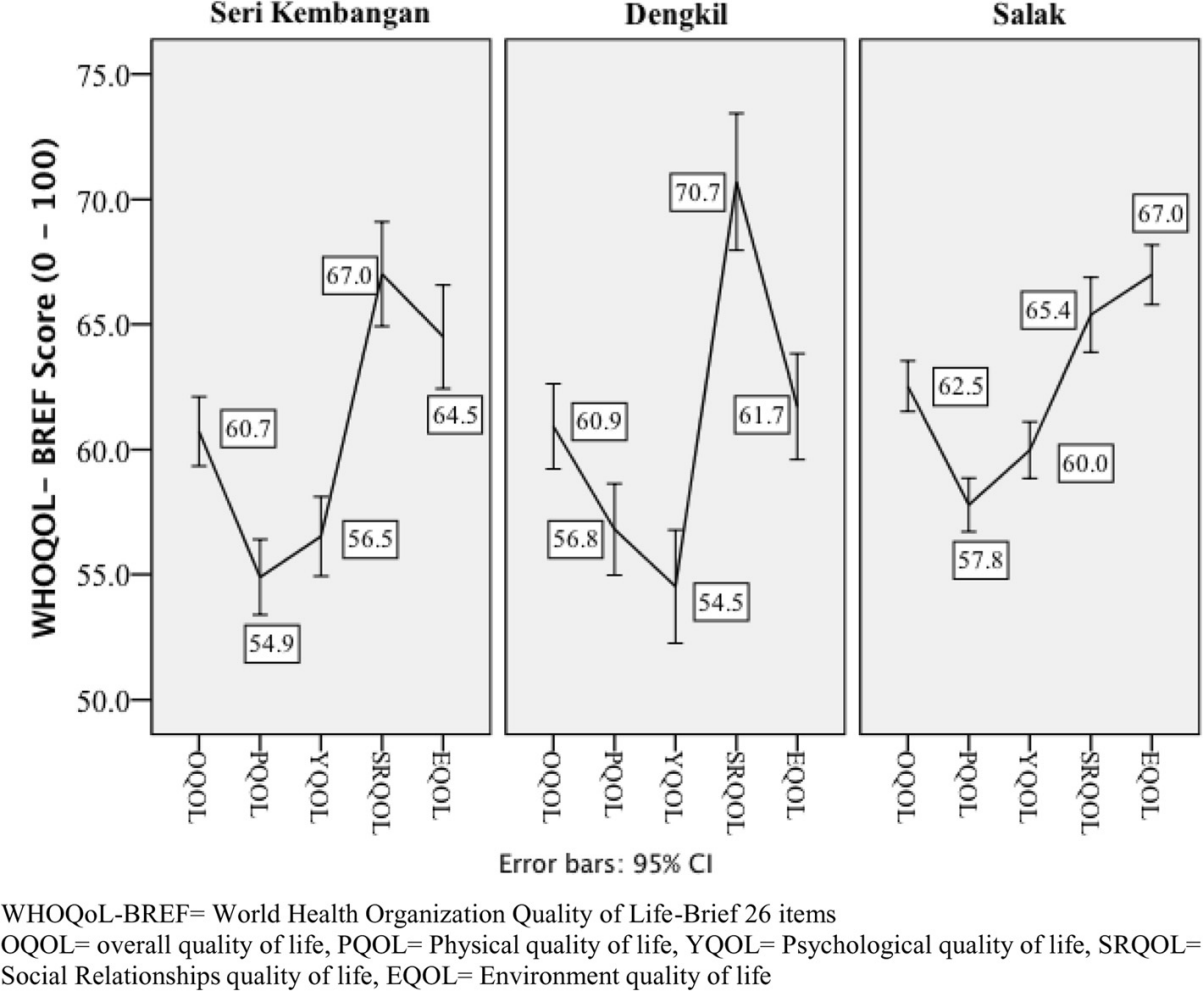
The WHOQOL-BREF mean (95 % confidence interval) of the total and domain score according to the three primary healthcare clinics in Malaysia

Based on the first two items of the WHOQOL-BREF, a majority of the adult patients with T2D at the primary care clinics had good HRQoL. About 60 % of the respondents had more than good overall HRQoL and were satisfied with their health (Fig. 1). The mean (SD) for OQOL, PQOL, YQOL, SRQOL and EQOL were 61.7 (9.86), 56.7 (10.64), 57.9 (11.73), 66.8 (15.01) and 65.3 (13.02), respectively. The patterns of distribution for WHOQOL-BREF domains in the three participating clinics were largely similar with PQOL and YQOL at the trough, and SRQOL and EQOL at the peak (Fig. 2). Patients from SL health clinic showed a significantly higher HRQoL. This association disappeared after the other socio-demographic variables were adjusted in the multivariable analyses (Table 2).

**Table 2:** The determinants of the health-related quality of life measured by the World Health Organization Quality of Life- Brief (WHOQOL-BREF)

	OQOL, *n* = 490		PQOL, *n* = 472		YQOL, *n* = 457		SRQOL, *n* = 560		EQOL, *n* = 535	
	Crude B (95.0 % CI)	Adjusted B (95.0 % CI)	Crude B (95.0 % CI)	Adjusted B (95.0 % CI)	Crude B (95.0 % CI)	Adjusted B (95.0 % CI)	Crude B (95.0 % CI)	Adjusted B (95.0 % CI)	Crude B (95.0 % CI)	Adjusted B (95.0 % CI)
(Constant)	-	71.36 (61.34, 81.38)	-	60.18 (48.9, 71.44)	-	69.38 (55.35, 83.41)	-	76.54 (65.10, 87.99)	-	69.34 (54.07, 81.61)
DDS score^a^	−0.18 (−0.22, −0.13)	−0.04 (−0.10, 0.02)	−0.13 (−0.17, −0.08)	0.01 (−0.06, 0.08)	−0.18 (−0.23, −0.13)	−0.12 (−0.20, −0.04)	−0.19 (−0.26, −0.12)	0.001 (−0.09, 0.09)	−0.22 (−0.28, −0.16)	−0.07 (−0.14, 0.01)
PHQ score^b^	−0.95 (−1.11, −0.80)	−0.81 (−1.02, −0.60)	−0.95 (−1.12, −0.77)	−0.87 (−1.11, −0.63)	−0.68 (−0.87, −0.48)	−0.42 (−0.69, −0.14)	−1.19 (−1.43, −0.95)	−1.21 (−1.52, −0.90)	−1.02 (−1.23, −0.80)	−0.74 (−1.02, −0.47)
Men	0.36 (−1.12, 1.83)	−0.76 (−2.42, 0.90)	1.70 (0.12, 3.29)	0.21 (−1.73, 2.14)	0.75 (−1.01, 2.50)	−0.19 (−2.42, 2.05)	−1.86 (−4.10, 0.38)	−2.70 (−5.06, −0.35)	0.90 (−1.05, 2.84)	−0.06 (−2.16, 2.03)
Malay^d^	1.94 (0.47, 3.42)	0.67 (−1.11, 2.45)	1.35 (−0.24, 2.94)	−0.70 (−2.78, 1.37)	2.58 (0.83, 4.33)	−0.57 (−2.96, 1.82)	2.58 (0.83, 4.33)	1.21 (−1.15, 3.56)	2.81 (0.87, 4.75)	2.60 (0.33, 4.88)
Age (year)	−0.06 (−0.13, 0.02)	−0.06 (−0.15, 0.03)	−0.14 (−0.21, −0.06)	−0.06 (−0.16, 0.05)	−0.14 (−0.23, −0.06)	−0.11 (−0.24, 0.01)	0.01 (−0.10, 0.12)	−0.12 (−0.24, −0.003)	0.05 (−0.05, 0.14)	−0.01 (−0.11, 0.10)
Diabetes duration (year)	-	-	−0.18 (−0.32, −0.04)	0.04 (−0.13, 0.21)	−0.18 (−0.34, −0.02)	−0.03 (−0.24, 0.17)	-	-	-	-
Health clinic^c^	0.93 (0.10, 1.76)	−0.95 (−2.32, 0.425)	1.44 (0.56, 2.32)	−0.46 (−2.02, 1.10)	1.95 (0.98, 2.93)	−0.78 (−2.56, 1.00)	-	-	1.41 (0.32, 2.50)	−1.00 (−2.64, 0.64)
Moslem^e^	-	-	-	-	−4.85 (−8.95, −0.75)	0.48 (−5.52, 6.32)	-	-	-	-
Religiosity^f^	3.93 (1.86, 6.00)	3.07 (0.46, 5.67)	−2.37 (−3.60, −1.14)	3.22 (0.27, 6.18)	5.29 (2.85, 7.73)	4.76 (1.15, 8.36)	-	-	-	-
Not married	-	-	-	-	−2.20 (−4.35, −0.06)	−2.11 (−4.62, 0.40)	-	-	−2.54 (−4.94, −0.15)	−2.82 (−5.39, −0.25)
Educational status^g^	-	-	3.25 (1.36, 5.14)	0.88 (−1.42, 3.17)	2.86 (0.76, 4.96)	0.76 (−1.83, 3.34)	-	-	-	-
Employment status^h^	1.41 (0.03, 2.80)	2.08 (0.43, 3.72)	2.93 (1.46, 4.41)	2.34 (0.46, 4.22)	2.59 (0.93, 4.24)	1.31 (−0.83, 3.45)	-	-	-	-
Exercise^i^	1.61 (0.69, 2.53)	1.31 (0.32, 2.29)	1.01 (0.01, 2.00)	1.08 (−0.04, 2.20)	-	-	2.61 (1.21, 4.00)	2.38 (0.93, 3.83)	2.05 (0.84, 3.26)	1.99 (0.69, 3.29)
BMI (kgm^−2^)	-	-	-	-	0.16 (0.0001, 0.31)	0.08 (−0.14, 0.29)	-	-	-	-
SBP (mmHg)	-	-	-	-	-	-	-	-	0.07 (0.01, 0.12)	0.07 (0.01, 0.13)
BP < 130/80 mmHg	-	-	−1.11 (−2.10, −0.13)	−1.43 (−3.37. 0.51)	-	-	-	-	-	-
Hypertension (Yes)	-	-	−2.44 (−4.37, −0.50)	−1.73 (−4.05, 0.58)	−2.83 (−4.95, −0.71)	−1.97 (−4.58, 0.63)	2.96 (0.22, 5.70)	2.57 (−0.29, 5.42)	-	-
CBG (mmol/L)	−0.30 (−0.50, −0.10)	−0.22 (−0.44, 0.001)	-	-	-	-	-	-	-	-
HDL-C (mmol/L)	−3.82 (−6.34, −1.31)	−2.71 (−5.49, 0.06)	−7.17 (−9.86, −4.48)	−4.51 (−7.58, −1.44)	−3.96 (−6.94, −0.99)	−1.42 (−4.91, 2.07)	-	-	-	-
Total-C (mmol/L)	−0.72 (−1.40, −0.05)	−0.19 (−0.91, 0.54)	-	-	-	-	−1.09 (−2.11, −0.08)	−0.63 (−1.65, 0.40)	−0.90 (−1.80, −0.01)	−1.04 (−1.94, −0.15)
Dyslipidaemia (Yes)	−2.18 (−3.71, −0.65)	−1.53 (−3.52, 0.46)	−1.98 (−3.61, −0.34)	0.32 (−1.96, 2.59)	−4.75 (−6.54, −2.96)	−3.14 (−5.78, −0.50)	-	-	−3.76 (−5.75, −1.76)	−2.78 (−4.48, −0.07)
MicroCx (Yes)	-	-	-	-	−3.28 (−6.66, 0.003)	−0.07 (−2.78, 2.64)	-	-	−4.34 (−7.99, −0.70)	−1.40 (−4.28, 1.48)
MacroCx (Yes)	−4.31 (−7.38, −1.23)	−4.98 (−8.56, −1.40)	−4.45 (−7.77, −1.13)	−3.50 (−7.36, 0.37)	−4.80 (−8.45, −1.14)	−4.45 (−9.42, 0.52)	-	-	−4.44 (−8.51, −0.38)	−4.38 (−8.30, −0.46)
OHA (Yes)	3.02 (0.41, 5.63)	1.56 (−1.40, 4.51)	3.42 (0.62, 6.21)	3.82 (0.43, 7.21)	-	-	5.37 (1.42, 9.31)	3.01 (−1.24, 7.26)	-	-
APA (Yes)	−2.73 (−5.10, −0.37)	0.13 (−2.56, 2.81)	−3.35 (−5.89, −0.81)	−1.53 (−4.65, 1.59)	−5.28 (−8.08, −2.49)	−0.15 (−3.73, 3.42)	-	-	-	-

Bs are unstandardized coefficients

*OQOL* overall quality of life, *PQOL* physical quality of life, *YQOL* psychological quality of life, *SRQOL* social relationships quality of life, *EQOL* environment quality of life, *AHA* anti-hypertensive agent, *APA* anti-platelet agent, *B* Coefficients, *CBG* casual blood glucose, *BMI* body mass index, *CI* confidence interval, *DDS* diabetes distress scale, *MacroCx* macrovascular complication, *MicroCx* microvascular complication, *OHA* oral hypoglycaemic agent, *PHQ* patient health questionnaire, *SBP* systolic blood pressure, *Total-C* total cholesterol

^a^Total DDS scores range from 17 to 102

^b^Total PHQ-9 scores range from 0 to 27

^c^Health clinic = Seri Kembangan (1), Dengkil (2), Salak (3)

^d^Non-Malay as reference

^e^Non-Moslem as reference

^f^Non-religious as reference

^g^Educational status = never (0), primary & secondary (1), tertiary and above (2)

^h^Employment status = unemployed (0), retired (1), employed (2)

^i^Exercise = no (0), at most three times per week (1), more than three times per week (2)

### Socio-demographic profile and quality of life

Male patients with T2D were generally experienced lower HRQoL except in PQOL and significantly so in SRQOL (Table 2). However, Malay patients had significantly better EQOL except in PQOL and YQOL (Table 2). Patients who were older and had diabetes for a longer duration had negative impacts on HRQoL in almost all of the sub-domains which was statistically significant with the SRQOL domain (Table 2). We observed that being religious was a socio-demographic variable that had the largest positive and independent effects on OQOL (B = 3.07), PQOL (B = 3.22) and YQOL (B = 4.76) (Table 2). Also, being employed as compared to those unemployed and retired, was a significant independent determinant of OQOL (B = 2.08) and PQOL (B = 2.34). Patients who exercised more frequently per week perceived higher OQOL (adjusted B = 1.31), SRQOL (adjusted B = 2.38), and EQOL (adjusted B = 1.99).

### Clinical parameters and quality of life

Having co-morbidities and diabetes-related complications would reduce WHOQOL-BREF scores (Table 2). Dyslipidaemia was shown to have significant independent negative impacts on YQOL (B = −3.14) and EQOL (B = −2.78). Although hypertension showed a positive effect on SRQOL (Table 2), MacroCx caused consistent negative and independent effects on HRQoL, OQOL (B = −4.98, 95 % CI −8.56 to −1.40) and EQOL (B = −4.38, 95 % CI −8.30 to −0.46).

In general, clinical biomarkers of disease condition and control had negative associations with HRQoL perception. The examples include HDL-C on PQOL (B = −4.51) and Total-C on EQOL (B = −1.04). Patients who were on OHA compared to those not taking any OHA were reported to have better HRQoL, and the most significant positive effect was seen on PQOL (Table 2).

### Diabetes-related distress, depressive symptoms and quality of life

The mean (SD) for total DDS-17 score was 37.1 (15.98) with 19.6 % (136/694) had moderate distress. Both higher DRD and DS were associated with lower HRQoL, especially on the YQOL (Table 2). DS showed more consistent, negative and independent effects across all the domains of HRQoL in comparison with DRD.

## Discussion

This study examined the HRQoL and its determinants in adults with T2D at the primary care level. WHOQOL-BREF was used instead of the disease-specific measure in order to determine the wider life impacts of T2D on patients which was rarely studied in the past. Nevertheless, in addition to the DS assessment a disease specific measure for distress, the DDS-17, was included in the present study.

It was disturbing to find that the mean score for PQOL was the lowest followed by YQOL. PQOL which represented physical functions, disability and needs showed that adult patients with T2D required much care for their pain, sleep and mobility. They also lacked of support from others to satisfactorily perform their daily activities. Physical problems such as pain, discomfort, and diet restrictions were also known to cause major problems in adult patients with T2D who were treated at primary care level in Singapore [50]. Perhaps more practical and psychological supports are needed to alleviate their perception on the burden of taking medication in order to improve their confidence when performing house chores and working. The findings on the YQOL domain revealed that adult patients with T2D experienced negative feelings (blue moods, despair, anxiety etc.) at primary care level which resulted in the inability to concentrate or enjoy a meaningful life. These two domains of poor physical and mental health had also been reported to have caused a median of 2 (IQR 0–10) unhealthy days in the past month among Irish patients with diabetes mellitus [51].

The SRQOL was the least affected among all the WHOQOL-BREF domains, and this was probably due to the prevalent of social support [52], culture [53], and family values such as filial piety in this country and region [54, 55]. However, these findings and values are not restricted to this study and region [25]. The perception on the quality of environment, living conditions, and access to transport and healthcare was categorised as moderate to good, and this was probably due to moderate ways of living and the culture of accepting current situations or fates. Although these personal attitudes and virtues could pose real barriers to diabetes care [56], it was uncertain whether having these qualities would help the patients to face life challenges and contribute to the high (about one third) responses of neither good nor dissatisfied in the first two items of WHOQOL-BREF.

### Socio-demographic profile and quality of life

Men with T2D were generally experienced lower HRQoL and significantly so in SRQOL except in PQOL. It was possible that these men were almost inherently the bread-winners of their families and were required to be out-and-about, and were less likely to complain or perceive themselves to be in need of basic help (just like men in most cultures). Hence, there was no association between these men and the physical domain of HRQoL [57]. In relation to men’s emotional ability [58], it was noted that they experienced the poorest SRQOL that required emotional skills for interpersonal relationships and the management of emotions. These findings were in contrast to the men with T2D from multi-ethnic backgrounds (non-Hispanic whites, African-Americans, Asian-Indians, and Hispanics) in Texas, United States (US) who were reported to have better diabetes-specific HRQoL compared to the women [55]. In another study, women who perceived living with diabetes as predominantly stressful intermingled with depressive feelings were required to be constantly vigilant about healthy eating, self-concern, and fatigue [3]. In this study, it was noted that HRQoL was better perceived by women because T2D had more adverse impacts on men, or the men’s perception could be limited by their emotional ability [58]. However, future studies are needed to confirm this and to examine the causes of low HRQoL among men with T2D.

Compared to other ethnic groups, Malay patients had a significantly better perception of their HRQoL in the univariable analyses. It was only statistically significant in the EQOL after the other covariates were adjusted and was negatively associated with the PQOL and YQOL domains (not reaching statistical significance). It was believed that this is closer to the actual states found in adults with T2D compared to the previous studies that reported positive association between Malays and their emotional and mental health status [59–61]. These past studies were limited by inadequate adjustments [59], loss of data quality due to the heterogeneity of the population (not specific to T2D), categorization of continuous outcome data and data-driven analysis [60, 61]. The Malays experienced better HRQoL, and this could be due to being more religious and having better social support network when faced with the external challenges of living with T2D [53, 60]. Nevertheless, self-care is patently a personal issue, and the impacts of changed life routines due to T2D are irrespective of ethnicity as evidenced by the non-significant differential effects of ethnicity on many HRQoL domains in the multivariable analyses.

After adjusting for co-morbidities and complications, this study showed the negative impacts of older age and having diabetes for a longer duration on HRQoL, especially on the social relationship domain. In a study involving Greek respondents, a generic SF-36 instrument was used on T2D patients in a rural setting, and it was discovered that women who were older, had diabetes for a longer duration and were unmarried were the predictors of impaired HRQoL [32]. Similar results were also reported from a Turkish setting where diabetes-specific HRQoL assessment tool was used (DQoL) [62], and T2D patients who were older than 40 year old, men, unmarried, had diabetes for more than 5 years, suffered from complications or prior hospitalization, and had HbA1c > 7 %, a significantly poorer overall HRQoL. It was possible that T2D patients who were older or had diabetes for a longer duration had higher cardiovascular risk, were prescribed more medications (and insulin), had more scheduled visits to different medical specialists, suffered from more co-morbidities, and complications which had reduced HRQoL [63]. It was unfortunate that although social care and support were available [25], the older patients found themselves inadequate for acceptable quality of life.

Amongst all the socio-demographic variables, being a religious person was noted to be the most potent determinant for HRQoL. The salutogenic effects of religiosity could be linked to the privileged relationship with the Higher or Supreme Being, stronger spirituality, and having a sense of purpose in life, social network and healthy lifestyles [64–66]. The ability to adhere to one’s religion and its way of life is defined as religiosity in this study. A patient must have had some physical agility that enabled him/her to attend places of worship and to participate in ceremonies or rites. Success in fulfilling these religious requirements might greatly ease any sense of guilt and improve spiritual well-being that could further induce a sense of harmony with the Supreme Being and psychological wellness [67, 68], which indeed is an internal resource for the self-management of T2D [69, 70]. Spirituality and spiritual support were the constructs that could relate to DS and affect HRQoL in T2D patients [71–73]. However, more research is needed to understand the independent associations of religiosity and the differential effects of the different religions, on HRQoL.

Also, it was reassuring to find that being employed and engaged in high frequency exercise per week contributed to better HRQoL. Most likely active employment provided economic security and social status, and the absence of either one or both factors would be detrimental to many aspects of human living and quality of life. Since active employment was generally equated to higher socio-economic status and education, the effects of these factors on HRQoL were also reported elsewhere [30, 32, 33]. In a more optimistic note, patients who were in active vocations might feel more fulfilled when completing their jobs or attending job-related engagements which could be lacking if they were in the retirement phase.

Exercise and HRQoL have a multi-level and multi-dimensional association, and the effects are beneficial to patients with T2D [74]. In a nationwide survey in the US, it was observed that a self-reported exercise was the only significant self-management behaviour to predict HRQoL after controlling the demographic and medical variables [26]. Physiologically, exercise or physical activity stimulates the release of endorphin that induces and facilitates a sense of elation, relaxation, and well-being [75]. Psychologically, when T2D patients adhere closely to the advice to exercise regularly, this may encourage a sense of compliance and harmonious relationship with their healthcare professionals and significant others [9]. In return, regular and increased frequency of exercise could also increase the social connectivity between the patients, their family members and friends, and also improve their physical health [76].

### Clinical parameters and quality of life

The negative and independent effects of MacroCx on OQOL were not unexpected because the consequences of IHD and stroke on patients’ daily life activities are known to many [29, 77]. In contrast, the more subtle and minimal the impacts of the early stages of MicroCx would explain the lack of its effects on HRQoL. Similar findings were also reported with diabetes patients at primary care from the Nordic countries [28], Greece [32] and Malaysia [61]. Although many different QoL measures were used in these studies, the results showed consistency and strong negative effects of MacroCx, especially coronary heart disease on HRQoL while weaker predictors were MicroCx, older age, women, lower education, lower income, normotensive and high HbA1c [28].

This study reveals that being diagnosed with dyslipidaemia could be more deleterious to YQOL than being diagnosed with hypertension. Although the effects of hypertension were expected to be more detrimental to the patients’ health than the effects of dyslipidaemia [32], it is intriguing to learn that T2D patients in this study perceived dyslipidaemia as worse than hypertension. It was possible to learn that dietary changes needed to control high cholesterol can be burdensome to some people and might be related to lower YQOL. Further study is needed to ascertain the possible reason adult patients with T2D chose dyslipidaemia and not hypertension on the YQOL. The negative effects of having dyslipidaemia and higher Total-C on EQOL might arise when patients’ desire for more physical activities were impeded. This might happen when patients were faced with non-conducive environment such as unhealthy weather, lack of sport facilities, and feeling unsafe when going out of the house. Past studies had also reported the prevalent association of high level clinical parameters such as HbA1c, blood pressure and lipid with lower HRQoL [28, 32, 33]. However, it was not uncommon to find the non-association between these biomarkers and HRQoL, or psychosocial variables such as social support, self-esteem and psychological well-being [78–80] in past literature. This discrepancy might be due to the different HRQoL measures used in the different studies [22].

It is difficult to hypothesise the negative effects of lower SBP, HDL-C, and OHA use on WHOQOL-BREF. For the BP target and HDL-C, it was possible to learn that that patients who had healthy diets and exercised regularly faced the physical and external barriers represented in the PQOL and EQOL domains. In the case of OHA use and better PQOL, it might be that the use of OHA had ameliorated the physical symptoms of uncontrolled diabetes mellitus leading to improved physical functioning of daily activities, sleep and concentration. Similar observations were reported in newly diagnosed Dutch patients in general practices who showed improved vitality scores and HRQoL following the first year treatment for their diabetes [81]. A recent systematic review suggested that a greater HRQoL could be gained if insulin or newer injectable agents (glucagon-like peptide agonists and analogues, amylin analogues) were added to the patient’s medication regimen rather than another OHA (e.g., adding sitagliptin or pioglitazone to metformin only) [82]. Unobserved determinants could probably explain the associations between treatment targets and HRQoL, particularly the SBP and its positive effects on the EQOL, such as the different geographic and social factors [83], dispositional optimistic personality and goal adjustment [84]. Also, being less critical to other people’s comments and having a could-not-care-less attitude towards one’s health [85, 86]. Alternatively, it is also possible that the indicators of the disease control and health were relatively less important as compared to MacroCx and/or MicroCx. the outcomes of poor disease control, on HRQoL [87] and hence resulted in the diverged associations between them.

### Diabetes-related distress, depressive symptoms and quality of life

Both DRD and DS were associated with lower HRQoL, especially on the YQOL. A Chinese study reported that emotional distress was the most important explanatory factor for quality of life which amounted to 28.7–53.8 % of the total variance [88]. However, this study showed that DS instead of DRD had a more consistent, negative, and independent effect across all the domains of HRQoL. Similar patterns of association were also reported by Carper et al. who stated that DS severity (assessed with Montgomery Asberg Depression Rating Scale) was associated with poorer HRQoL (measured with Quality of Life Inventory), specifically on the achievement, psychosocial growth and environment domains while DRD was associated with poorer HRQoL on the achievement domain [89]. The results of the present study suggested that DRD and DS were related, but were distinct constructs [47] associated with the various aspects of HRQoL that were beyond demographic and clinical factors.

Sundaram et al. reported the pervasive effects of DS among adult patients with T2D on a number of QoL measures that included the generic health status (12-item Short-Form Health Survey [SF-12] and EQ-5D) and diabetes-specific QoL (Audit of Diabetes Dependent Quality of Life) [90]. The degree of diabetes-specific QoL perception was reported to be associated with the severity of DS (*r* = 0.503; *p* < 0.001) among Brazilian patients with T2D [78]. Similarly, DS (measured with SF-12 Mental Component Score) among the elderly German patients with T2D was one of the independent predictors for HRQoL [91]. Besides DS, other mental disorders such as anxiety and schizophrenia had also been reported to be significant predictors for poorer diabetes-specific QoL [92].

These findings confirm the past reports on the prevalent and intimate relationships between the domains of HRQoL and emotions (DRD and DS), psychological well-being, and social functioning in adult patients with T2D [28]. By providing effective psychological support for DRD and DS, HRQoL may benefit to a large extent. Since evidence showed that DRD had preceded DS [93], and that DRD was both milder and more common in primary care as compared to DS [94], it would be a wise therapeutic and preventive opportunity to intervene for DRD in order to reduce the DS and its distal adverse effects and complications. An intervention for a relatively non-complicated psychological disorder such as DRD could probably be addressed with a less complex programme and competently delivered by the paramedics.

### Limitations and strengths

Adopting a generic measure for HRQoL would cause some limitations and bring different interpretations. It was possible for T2D patients to report high levels of HRQoL in general, but poor HRQoL meant the patients were impaired by diabetes [95]. During clinical consultations, when opportunities arise to tailor the treatments of each T2D patient for the purpose of improving the patient’s HRQoL, it is essential to differentiate between the general QoL and disease-specific HRQoL because patient’s needs might go unnoticed if the general well-being or QoL are the only outcomes measured, and if it appeared to be good [96].

The strength of this study includes the relatively large sample size with high response rate, and it also represents the study population to the study domain from the aspects of socio-demographic characteristics [97]. Another important strength of the present study is the use of a validated and specific measure of DRD.

## Conclusions

The majority of adult patients with T2D at a primary care setting had a good overall HRQoL. The independent determinants for HRQoL concurred with many of the past studies. In addition, DRD was reported to have negative effects on HRQoL. Meanwhile, religiosity had positive influences on HRQoL. Adult patients with T2D who were men, non-Malay, unmarried, unemployed, from older age group, had longer duration of diabetes, dyslipidaemia, MacroCx, did not engage in frequent exercises per week, had higher levels of Total-C, who experienced DRD and DS had lower HRQoL needed additional supports. Timely and less complex psychological intervention at primary care level could be prioritised for the right and most needy adult patients with T2D to improve their life experiences and HRQoL.

## Abbreviations

AHA: Anti-hypertensive agents
BMI: Body mass index
DDS-17: 17-items diabetes distress
DK: Dengkil health clinic
DRD: Diabetes-related distress
DS: Depressive symptoms
EQOL: Environment quality of life
HDL-C: High density lipoprotein-cholesterol
HRQoL: Health-related quality of life
LDL-C: Low density lipoprotein-cholesterol
LLA: Lipid-lowering agents
MicroCx: Microvascular complications
MacroCx: Macrovascular complications
MREC: Medical Research Ethics Committee, Ministry of Health, Malaysia
OHA: Oral hypoglycaemic agents
OQOL: Overall quality of life
PHQ-9: 9-items Patient Health Questionnaire
PQOL: Physical quality of life
QoL: Quality of life
SK: Seri Kembangan health clinic
SL: Salak health clinic
SRQOL: Social relationships quality of life
TG: Triglyceride
T2D: Type 2 diabetes
WHOQOL-BREF: World Health Organization Quality of Life- Brief
YQOL: Psychological quality of life
